# Injectable Nanocurcumin-Formulated Chitosan-g-Pluronic Hydrogel Exhibiting a Great Potential for Burn Treatment

**DOI:** 10.1155/2018/5754890

**Published:** 2018-05-13

**Authors:** Le Hang Dang, Thi Hiep Nguyen, Ha Le Bao Tran, Vu Nguyen Doan, Ngoc Quyen Tran

**Affiliations:** ^1^Institute of Research and Development, Duy Tan University, Da Nang 550000, Vietnam; ^2^School of Biotechnology, International University, Vietnam National University, Hochiminh City 70000, Vietnam; ^3^Biomedical Engineering Department, International University, Vietnam National University, Hochiminh City 70000, Vietnam; ^4^University of Science, Vietnam National University, Hochiminh City 700000, Vietnam; ^5^Graduate School of Science and Technology, Vietnam Academy of Science and Technology (VAST), 1A TL29, District 12, Hochiminh City 700000, Vietnam; ^6^Institute of Applied Materials Science, Vietnam Academy of Science and Technology (VAST), 1A TL29, District 12, Hochiminh City 700000, Vietnam

## Abstract

Burn wound healing is a complex multifactorial process that relies on coordinated signaling molecules to succeed. Curcumin is believed to be a potent antioxidant and anti-inflammatory agent; therefore, it can prevent the prolonged presence of oxygen free radicals which is a significant factor causing inhabitation of optimum healing process. This study describes an extension of study about the biofunctional nanocomposite hydrogel platform that was prepared by using curcumin and an amphiphilic chitosan-g-pluronic copolymer specialized in burn wound healing application. This formular (nCur-CP, nanocomposite hydrogel) was a free-flowing sol at ambient temperature and instantly converted into a nonflowing gel at body temperature. In addition, the storage study determined the great stability level of nCur-CP in long time using UV-Vis and DLS. Morphology and distribution of nCur in its nanocomposite hydrogels were observed by SEM and TEM, respectively. *In vitro* studies suggested that nCur-CP exhibited well fibroblast proliferation and ability in antimicrobacteria. Furthermore, second- and third-degree burn wound models were employed to evaluate the in vivo wound healing activity of the nCur-CP. In the second-degree wound model, the nanocomposite hydrogel group showed a higher regenerated collagen density and thicker epidermis layer formation. In third degree, the nCur-CP group also exhibited enhancement of wound closure. Besides, in both models, the nanocomposite material-treated groups showed higher collagen content, better granulation, and higher wound maturity. Histopathologic examination also implied that the nanocomposite hydrogel based on nanocurcumin and chitosan could enhance burn wound repair. In conclusion, the biocompatible and injectable nanocomposite scaffold might have great potential to apply for wound healing.

## 1. Introduction

Skin wounds are inevitable in life; thus, functional recovery after injury remains the principal goal of tissue engineering research [1–3]. After the skin has been getting either acute or chronic injuries, the body initiates the process comprised of four phases: hemostasis, inflammation, proliferation, and remodeling at the injured site, in which heal injured tissue can heal and can reconstitute its barrier function [4, 5]. However, in cases of large injuries, second- or third-degree burn, due to the long-term treatment, the natural process of wound healing is not perfect resulting into physical and psychological scars and cause chronic disabilities or even a significant cause of morbidity and mortality [3, 6]. A factor contributes to fail the self-healing properties of skin is an extended period of the wound repair process due to the prolonged presence of oxygen free radicals [7], which is generated by high concentration of reactive oxygen species (ROS). More concentration of oxygen free radical leads to the more oxidative stress causing inhabitation of optimum wound healing [8]. Therefore, the reinforcement of free radical scavenging activity using antioxidant therapy [9, 10] in the early stage can significantly promote the healing process of wound. According to the report of Wertheim et al. [11] and Sevitt [12], another factor is related to the extension of microorganisms beyond the eschar into viable tissue. Currently, numerous reports have been supporting the fact that the prolonged time would increase susceptibility to infection in burn patients [4, 6, 13]. At the burn wound surface, the presentation of death tissue or eschar tissues provides not only a rich nutrient for the growth of bacteria [14], fungi [15], and virus [16, 17], but also the protection for them from the host immune cells and the locally applied antimicrobial drugs [18–20]. Beyond the invasion of microorganism that can release toxic in the tissue layer, bloodstream resulting in the elongation of inflammation, faulty re-epithelialization and deficient matrix remodeling, as follows, can disorder the healing process [4, 19, 20].

Wound dressing has evolved according to the pathogenesis of different wounds [19]. A well-formed wound dressing has the ability to provide hydration, absorb the excessive exudates, act as a barrier against microorganisms, avert per-wound skin, be nontoxic, reduce maxima pain during application and withdrawal, and promote wound healing [21, 22]. Various dressing subtypes are introduced in the market such as transparent film dressings [23, 24], foam dressing [25, 26], hydrogel [27, 28], and and so on. Although there is lack of the information in comparative effectiveness data among dressing subtypes, there are enough evidences in which hydrogel is strikingly impressive [6, 21, 27]. Hydrogel is defined as a complex 3D structure of hydrophilic cross-linked polymers providing a flexible structure with porous architecture [29, 30]. Therefore, hydrogel can provide dynamic equilibrium between the excessive exudates and donate moisture to the wound surface, consequently assisting autolytic debridement and optimizing pH, temperature, and moisture [6, 31]. Hydrogel allows for gaseous exchange leading to the higher oxygen tensions which can promote a greater enviroment for cellular metabolism, thus promoting healing [21, 22, 32].

A standout amongst the most plenteous hydrogel in tissue designing application is the chitosan hydrogel [33, 34]. Chitosan is a natural polymer most broadly utilized as a part of wound regeneration because of its many focal points including biocompatibility, biodegradability, hemostatic activity, and antibacterial properties [33, 35]. Chitosan is structurally similar to naturally occurring glycosaminoglycan and is degradable by enzymes in humans [36, 37]. It is gotten from chitin [38], which is found in arthropod exoskeletons. Chitosan is a linear polysaccharide of (1–4)-linked d-glucosamine and N-acetyl-d-glucosamine residue from chitin. Clinical information of a human report using a chitosan film as a wound dressing indicated effective adherence, hemostasis, mending, and re-epithelization of the injury [37, 39].

Pluronic F-127 is a temperature-stimulus hydrogel highly recommended in pharmaceuticals as a carriage for various courses of administration including oral, topical, intranasal, vaginal, rectal, ocular, and parenteral [40, 41]. Pluronic F-127 has a decent solubilizing limit and high biocompatibility and is, along these lines, considered a decent medium for drug delivery frameworks [41]. It is more soluble in low temperature than in room temperature because of expanded solvation and hydrogen bonding at bring down temperatures. The aqueous solutions have the ability of reverse thermal gelation, which means that it is liquid at low temperatures and gel when warmed or at room temperature. Pluronic F-127's ability to gel at higher temperatures allows it to transform from an applied cold solution to solid gel form when heated by body temperature. Upon its application onto skin or injection into a body cavity, the gel serves as a solid artificial barrier and a sustained release depot. In addition, Pluronic is FDA endorsement for wound dressing in light of the fact that pluronic can go about as a cleaner to expel the debris or death tissues which generate from the wound [40, 42].

In the current trend in tissue engineering, in order to enhance healing process of wound, wound dress should be synthesized in the combination of the active gradients such as growth factor proteins (EFG, FGF-2, etc.,) [43–45], bioactive compounds (curcumin, quertince, etc.,) [8, 46, 47], or antibacterial drugs (silver, ciprofloxacin, etc.,) [48–51]. With the aim of decreasing the problems related to prolonged healing time, but noncytotoxic activities that have specific destructive action on certain cells and nonresistance of microorganism, utilize of natural medications in wound healing of burn injuries have been satisfactorily [8, 46]. Among various suitable natural medications for this purpose, curcumin, an active substance in the Turmeric (*Curcuma longa L*.), has shown multiple pharmacological properties such as anti-inflammatory [52, 53], anti-infectious [54], and antioxidation activities [54, 55] and stimulation of fibroblast proliferation [53, 56]without toxicity even at high doses [56]. Despite a good ideal for wound healing, the therapeutic efficacy of Cur is still limited due to its poor bioavailability and poor stability [57, 58]. In order to achieve these remained dilemmas, a formulation of the thermal sensitive nanocurcumin-encapsulated chitosan-g-pluronic hydrogel was developed in our laboratory [59]. The formulation of nanocomposite showed an initial structural stability, a favorable environment for cell in growth and accessible modulation as transdermal drug delivery. Extension of the preceding study, some *in vitro* experiments with human fibroblast cells (hFcs) and their behaviors in *in vivo* second/third burn degreed models are disclosed herein.

## 2. Materials and Methodology

### 2.1. Material

The hydrogel and nCur-CP nanocomposite was synthesized and characterized as previously [59]. All the solvents in this study (absolute ethanol, acetonitrile, methanol, etc.,) were purchased from Sigma (USA) or Schalaurs (Spain).

#### 2.1.1. Animal and Ethical Guidelines

The healthy male Musmusculus var. Albino mice were purchase from Pasteur Institute, Ho Chi Minh City. The Animal Care and Use Committee of the University of Science, Vietnam National University at Ho Chi Minh City (Registration Number 10/16-010-00) approved all procedures.

### 2.2. Characterization of nCur-CP

To observe the morphology of nCur-CP, the freeze-dried sample was fractured carefully, and then the interior morphology of the hydrogels was visualized by using a scanning electron microscope (JEM-1400). The free-dried nCur-CP was dissolved into water and then created a thin film in the disk to determine the morphology of curcumin nanoparticles through transmission electron microscopy (JSM 7401F).

The biodegradation of nCur-CP was investigated by testing their water absorbance as well as their erosion behavior. 1 ml sample of CP (15% w/v), nCur-CP (15% ww/v), and Pluronic F127 (18% w/v, the concentration at which pluronic can form gel) were placed into a water-bath at 37°C. After gel stage formed in each vial, the vials were weighed to calculate the mass of gels that were used as the initial values (Wi). 10 ml PBS (pH = 7.4, 37°C) was added into each vials and incubated at 37°C. After removing the supernatant, the remaining hydrogel sample was recovered, dried, and weighed at a predetermined time point (*n*=3). The swollen level of the hydrogels was determined using the following formula:

(1) $$\begin{array}{c} \text{Mass swelling}\left(\%\right)=\frac{W_{\mathrm{st}}}{W_{i}}\times 100\%, \end{array}$$

where *W*_st_ is the weight of the swollen hydrogel at the specific time.

The percentage of erosion of hydrogels was calculated as:

(2) $$\begin{array}{c} \text{Remaining dry mass}\left(\%\right)=\frac{W_{\mathrm{dt}}}{W_{\mathrm{di}}}, \end{array}$$

where *W*_dt_ is the weight of dried mass at a predetermined time, while *W*_di_ is the weight of the dried mass at the initial.

### 2.3. Storage Study

The stability of freeze-dried optimized curcumin nanoparticulate formulation was checked at 2 different conditions: refrigerated condition (4–8°C) and ambient condition (room temperature (RT)) at 4 different time points (0, 1, 4, and 24 weeks). After freeze-drying, 3 ml freeze-dried curcumin nanoparticulate formulations in glass injection vials were stoppered with rubber stoppers and sealed with aluminum using crimper. The sealed vials were then placed in individual cardboard boxes (secondary containers), charged for refrigerator, and kept under ambient laboratory condition. At specified intervals, the samples were withdrawn and following tests were performed: appearance of freeze-dried product, reconstitution score, appearance of reconstituted product, particle size (dynamic light scattering (DLS) by using SZ-100 Nanoparticle Analyzer, Horiba, UK), and drug content (high-performance liquid chromatography (HPLC) by using Agilent C18 with running program: acetonitrile: acid water (pH = 5.0) = 45 : 55 in volume). Reconstitution was done with the original volume of purified water to restore the curcumin concentration.

### 2.4. In Vitro Evaluation of the Injectable nCur-CP Materials

#### 2.4.1. Antibacterial Activity

The antimicrobial activity of curcumin was tested against *Escherichia coli* (ATCC8739), *Salmonella typhimurium* (ATCC 14028), *Pseudomonas aeruginosa* (ATCC27853), and *Staphylococcus aureus* (ATCC 6538) obtained from Department of Biochemistry, Faculty of Biology and Biotechnology, University of Science, Vietnam National University, Ho Chi Minh City. Nutrient agar was used to culture the test bacteria. Each strain was transferred from stored slants at 4°C to 10 mL of nutrient broth tube and cultivated overnight at 37°C. The bacterial cultures were then diluted in sterile LB solution and adjusted to a cell suspension of 10^6^–10^7^ colony forming unit (cfu)/mL using a UV spectrophotometer at 660 nm. There are 4 variables in this test: nC-CP, hydrogel, curcumin solutions, and antibiotics chloramphenicol as the control. The concentration of raw curcumin was approximate to the concentration of curcumin in hydrogel composite nCur-CP for zone inhibition testing. A welldiffusion method was used to assay the antibacterial against test strains on nutrient agar. A total of 100*μ*L of diluted inoculum (10^6^–10^7^ cfu/mL) from organism suspensions was spread on the surface of the plates and allowed to solidify. Three wells were cut out with the help of a well borer under aseptic conditions on the agar medium. They were filled with 20*μ*l of each variable. The plates were incubated for 24 h at 37°C. The antimicrobial activity was evaluated by measuring the diameter zone of transparent inhibition against test microorganisms.

MIC of curcumin and nanocurcumin was tested by the agar dilution method. The stock solution of all variables was prepared. For curcumin, a similar aqueous stock solution could not be prepared because curcumin is completely insoluble in water. Therefore, the stock solution was prepared by dissolving in DMSO. To flasks containing 20 mL of melted agar, different concentrations (0.08–4*μ*g/mL) of curcumin (DMSO), (0.75–37.5 mg/mL) hydrogel, and its nanocomposite (water) solutions were added separately. An equivalent amount of DMSO was used in the control plates, and they were then left to solidify. A total of 100⁡*μ*L of culture was inoculated under aseptic conditions, and the plates were incubated at 37°C for 24 h in the case of bacteria. Each experiment was performed in duplicate and repeated 3 times. The MIC was reported as the lowest concentration of curcumin capable of completely inhibiting the growth of each bacterial being tested.

#### 2.4.2. Cell Culture

Human foreskin fibroblasts (HFF-1; SCRC-1041TM; USA) were used for cytotoxicity evaluation and cell proliferation. The HFF-1 cells were cultured in Dulbecco's modified Eagle medium (DMEM; Gibco BRL, USA) supplemented with 10% (v/v) fetal bovine serum (FBS; Gibco BRL, USA), 100 U/ml penicillin G (Gibco BRL, USA), and 100*μ*g/mL streptomycin (Gibco BRL, USA). The cells were cultured in 150 mm Petri dishes at 3 × 10^5^ cells in a humidified atmosphere of 5% CO_2_ at 37°C. The culture medium was replaced every three days. Once cells reached 80% confluence, they were detached by using Trypsin-EDTA (Gibco BRL, USA), resuspended in DMEM, and used for further study.

HFF1-cell proliferation was evaluated on 2D tissue culture control, hydrogel, and nanocomposite materials containing 55.12 ppm curcumin. Hydrogel and its nanocomposite were obtained by reconstitution of freeze-dried hydrogels and nCur-CP with DMEM. The solution was maintained on ice for about 5 to 6 h with periodic vortexing to ensure complete dissolution. A volume of 100*μ*L of hydrogel and its nanocomposite were pipetted into the wells, respectively, which were then heated to 37°C for 2 minutes to induce gelation. HFF1 cells (3 × 10^5^) in 20*μ*L fully supplemented DMEM were seeded into uncoated tissue culture control, hydrogel, and nanocomposite hydrogel-precoated wells. Fully supplemented DMEM medium was added 1 h after seeding. After 12 hours of incubation, these cellular materials were washed with PBS three times. Cell viability was evaluated by commercially available live/dead viability/cytotoxicity kit (Life Technologies Korea LLC, Seoul, Korea); and HFF1- cell proliferation was measured with Alamar Blue (Sigma-Aldrich, St. Louis, MO, USA) after 40 h culture. The results were observed using fluorescence microscope (TE2000, Nikon, Seoul, Korea) equipped with a digital camera. The cell number was determined from calibration curves generated with known numbers of HFF1 cells.

### 2.5. In Vivo Evaluation of Burn Wound Healing in Animal Model

The experiment was conducted at the Laboratory of Department of Physiology and Animal Biotechnology under permission of the ethical committee for biomedical research of University of Science, Vietnam National University, Ho Chi Minh City, Vietnam. The mice were anesthetized by intraperitoneal ketamine (100 mg/ml) and xylazine (20 mg/ml) with dosage of 0.2 ml/100 g body weight. The dorsal skin of the animals was shaved and cleaned with 70% ethanol and 1% polyvinylpyrrolidone iodine. In order to get the right level of burn, a cylindrical shaped stainless steel cup with radius of 0.5 cm was placed in hot water (∼100°C) and then on the backs of mice and held for various time (3 s, 5 s, 10 s, and 20 s). After 2 or 3 hours, these skins were collected and processed with haematoxylin and eosin staining. Based on the criteria of Tiwari [60], the second-degree burn and third-degree burn models were applied for further study.

In the study of wound healing property, animals were divided in to three groups, each group comprising of three mice:

- Group I: control (nontreatment).
- Group II: standard treatment group (the standard method applied to treat second-degree burn with Biafine cream/third-degree burn with Silvirin).
- Group III: nCur-CP group.

The second-degree burn was studied in 14 days whereas it took 22 days for third-degree burn. Progression decrease in the wound size was monitored periodically by measuring the distance between 2 opposite outside edges of the wound margin, using a vernier caliper (Figure 8). Two measurements approximately 90° from each other were obtained; the largest distance was used as one of the measurements. During the measurements, mice were fixed on a table. Wounds were digitally photographed on 0, 2, 4, 6, 8, 10, 12, and 14 days after wounding second-degree burn and continuously on 16, 18, 20, and 22 days after third-degree burn wounding, with maintaining constant optical zoom.

After day 14, the tissues from second-degree burn model were routinely processed by standard procedures and stained with hematoxylin and eosin (H&E) and Masson's trichrome. In third-degree burn model, skin biopsies of wounds were collected at the 7th, 14th, and 22nd day of wound treatment.

Using ImageJver 1.41o, the Masson-trichrome-stained tissue sections were used measure the collagen signal in the entire granulation tissue area at a constant threshold.

### 2.6. Statistical Analysis

Values were expressed as the mean ± standard error. The statistical differences between the means were determined using 1-way ANOVA followed by Dunnet's test in terms of particle size, PDI, content of curcumin, antibiotic testing, and so on. The data concerning cell viability and wound healing surface were statistically analyzed using two-way ANOVA test and Tukey multiple comparison test. A *p* value of < 0.05 was considered statistically significant.

## 3. Results

### 3.1. Characteristic of nCur-CP Nanocomposite Hydrogel

For the preparation of the injectable nanocomposite hydrogel, some preliminary experiments were proposed [59]. The phase transition temperature of CP in water was in the range from 30°C to 35°C when the concentration of the chitosan modification product is 15% w/v. Secondly, curcumin in solvent (DCM: ethanol = 30 : 70) was dropped wise into aqueous CP and started ultrasonication with Hielscher UP200Ht, at 50 Wt per mL of solution. After 10 min of sonication, curcumin was separated from solution by centrifugation and resuspension in DI water. By analyzing rheological properties of nanocomposite hydrogel at 15% w/v [59], there was no significant change in transition temperature point of this system, about 35°C, within a range in which the purpose of the present invention is met.

In the reports, some morphological and biological characterizations were studied towards tissue regeneration. Morphology and size distribution of curcumin nanoparticles were investigated by TEM (JSM 7401F) and SEM (JEM-1400). SEM image (Figure 2) showed that the inner pores of the CP are interconnected with irregular shapes as well as the embedment of nCur to form a relatively homogeneous composite. In other words, morphology of nCur-CP exhibited porous structure. Moreover, the regular dispersion of the curcumin nanoparticles on the surface of the CP hydrogel (Figure 2) exploited at the higher magnification of SEM imaging (×10, 000) showed that nCur exhibited a spherical morphology, similar to the TEM image (Figure 2). In addition, nCur were obtained with edges from 20 nm to 50 nm with SEM imaging and TEM imaging, whereas the overage size of 55.9 ± 1.86 nm was determined in light scattering experiments (SZ-100–Horiba).

Controlling the excess wound exudate plays a critical role in wound healing, especially in burn wounds. In the exudate, the amount of proteinases is very high [4–6] causing considerable problems such as discoloration of the wound bed or excoriation at the wound margins and in the surrounding skin. The higher level of wound exudate, when left in contact with the skin, can cause maceration even when the fluid constituents are relatively benign. Moreover, this fluid contains large amount of nutrient that promotes the entry of bacteria into the wound-bed resulting in enhancement the risk of infection. Therefore, in order to prevent a clinical problem of exudate, the ideal hydrogel should absorb all wound exudates and fluids on the wound surface. The fluid uptake ability of nCur-CP (15% w/v) was compared with CP (15% w/v) and Pluronic F127 (18% w/v) flowing the weight change or mass swelling of hydrogel (water uptake) as the function of time in PBS solution (pH = 7.4) at 37°C. It was noticed that although all formulations showed rapid swelling initially after 1 h and up to 4 hrs due to the porous nature of the hydrogels offering large surface area allowing rapid uptake of the solvent, the levels of swollen hydrogel were significant different in three samples (*n*=3, *p* < 0.05). nCur-CP exhibited the best water uptake ability; this hydrogel had gained weight to 276.53 ± 11.35% of that of the initial and no significant change regardless of time flow (from 4 h to 50 h). CP hydrogel with the same concentration showed the trend of swelling up as similar as CP containing nCur. However, Pluronic F127 hydrogel with 18%w/v eroded rapidly after reaching the maximum swollen stage under the same conditions. Furthermore, the mass erosion behaviors of three samples were also investigated by comparison of the dry mass at specific time point. As given in Figure 3(b), the mass erosion of 3 samples were different in PBS (pH = 7.4) at 37°C (*n*=3, *p* < 0.05). nCur-CP exhibited a wide range of erosion rates from several days, while CP showed slight erosion. The highest rate mass erosion in aqueous solution was in Pluronic F127 hydrogel at 18%. These results supported to swelling behavior of three samples in the same condition. The different between nCur-CP and CP can be explained through the rheological study through the temperature oscillation testing. Although the temperature at which phase transition occurs was the same (at 35°C), storage modulus (G′) and loss modulus (G″) were different (data arenot shown). The value of G′ and G″ at the transitional temperature of the nCur-CP were 1.5 times as in CP hydrogel with the same concentration (15% w/v). The network in the nCur-CP was more complicated or higher cross-linking density when compared with CP without nCur.

### 3.2. Storage Stability of Curcumin Nanoparticles

After 24 weeks of storage at low temperature (in range 4°C–8°C) and ambient conditions, no change was observed in the physical appearance or colour of all the stored stability samples. The changes in particle size (Figure 4(a)), PDI (Figure 4(b)), and the concentration of curcumin (Figure 4(c)) during the storage time at different conditions were investigated in this study. At RT, statistically significant differences in particle size were noticed after 4 and 24 weeks. Overall significant increase in the particle size in RT (from 55.9 ± 1.86 to 85.9 ± 4.5 nm) and small increase trend in cool condition (from 56.5 ± 3.7 nm to 61.2 ± 3.8) were noticed after 24 weeks at all the storage conditions, but their polydispersity index remained below 0.2 which reflects relatively homogeneous particle. Following the analysis of ANOVA, there is no statistically significant change in the content of curcumin after 24 weeks of storage at low temperature and ambient conditions in comparison to initial curcumin.

### 3.3. In Vitro Evaluation of nCur-CP Nanocomposite Hydrogel

#### 3.3.1. Antibiotic Ability

The antimicrobial activity of nCur-CP was tested against *Escherichia coli* (ATCC8739), *Salmonella typhimurium* (ATCC 14028), *Pseudomonas aeruginosa* (ATCC27853) and *Staphylococcus aureus* (ATCC 6538) by using the Kierby–Bauer disc diffusion method. In the present study, hydrogel CP, hydrogel composite nCur-CP, and antibiotics chloramphenicol were used to determine antibacterial activity (Figure 5). The concentration of raw curcumin used in testing was equal with the concentration of curcumin in the nCur-CP sample. The zones of growth inhibition around the disks were measured after 18 to 24 hours of incubation at 37°C for bacteria. The sensitivities of the microorganism species to samples were determined by measuring the sizes of inhibitory zones (including the diameter of disk) on the agar surface around the disks, and values ≤6 mm were considered as not active against microorganisms. Commercially available antibiotic chloramphenicol was found to be the same effective against 4 bacterial strains in this test. nCur-CP materials were found more effective against *P. aeruginosa* (27 ± 1.2 mm), *S. aureus* (27 ± 0.5 mm), and *E. coli* (24 ± 0.3 mm), whereas less effective against *S. typhi* (20 ± 0.5 mm). Also, it was the trend of hydrogel which were most effective against *P. aeruginosa*, *S. aureus*, and *E. coli* and less effective against *S. typhi*. However, raw curcumin samples were the most effective against *S. aureus* (20 ± 1.0 mm) while less effective against *E. coli* (14 ± 0.3), *P. aeruginosa* (11 ± 0.5 mm), and *S. typhi* (9 ± 0.2 mm). In addition, by comparison using ANOVA one way for each bacterial strains, the combination of curcumin and hydrogel showed maximum efficiency in all tests, which is significantly different when compared with other treatments (*p* < 0.05).

Minimum inhibitory concentrations (MICs) are defined as the lowest concentration of an antimicrobial that will inhibit the visible growth of a microorganism after overnight incubation. MIC of nCur-CP for *S. typhi*, *E. coli*, *S. aureus*, and *P. aeruginosa* was 4.5 (0.48 ppm curcumin), 3.0 (0.32 ppm curcumin), 1.5 (0.16 ppm curcumin), and 0.75 (0.08 ppm curcumin) mg/mL, respectively, compared to 4, 1.6, 0.96, and 2 ppm for raw curcumin (Table 1). For all experiments in MIC, the antimicrobial activity was within the expected ranges of zone of inhibition. Taken together, the results indicated that the selected Gram-positive bacteria had higher sensitivity than the selected Gram-negative bacteria in raw curcumin samples. This could be due to differences in their cell membrane constituents and structure. It is known that Gram-positive bacteria contain an outer peptidoglycan layer, while Gram-negative bacteria contain an outer phospholipidic membrane, both of which undergo different types of interaction when encountered by curcumin. In case of nCur-CP and CP hydrogel, the incorporation of chitosan, which has broad-spectrum antibacterial activities, might disrupt the surface charge on membrane of Gram-negative bacteria. Notably, with the help of chitosan via electrostatic interaction with the outer membrane of both negative and positive bacteria, curcumin particles effectively enter into bacterial cells when compared with CP hydrogel without curcumin.

#### 3.3.2. Biocompability Test

As fibroblast cells are key factors in wound healing [63], we chose to use them for our experiments. The proliferation rate of HFF1-cells was compared among cultures on 2D control, hydrogel, and nCur-CP containing 55.12 ppm curcumin.  Figure 6(a) shows that HFF1 cell proliferation rate was lower for cultures on hydrogels as compared with cultures on control environment (*p* < 0.05). The number of cells on nCur-CP was greater than all other conditions (*p* < 0.05). Besides, cells in pure hydrogel maintained a short spindle-shaped morphology and with little evidence of interaction with the surrounding matrix, whereas the spindle-shaped morphology was observed in both nCur-CP and control. The observed morphology was consistent with a study in mouse fibroblast cells culturing on hydrogel (remained short spindle shape in vitro over a 72 h culture (data was not shown)) and some studies [64, 65]. This may be due to the interaction of chitosan on cell membrane, which tends to weaker cell membrane; consequently, fibroblast cells are less spreading. In aspect of nC-CP, after induced competent cell membrane by chitosan, nanocurcumin, that is known as the factor that can increase migration and proliferation of fibroblasts [66, 67], is released into the cell resulting in the greatest proliferation rate as compared with control sample. Nevertheless, because of the self-florescence of curcumin [68], we cannot use live/dead assay to evaluate the fibroblast cell on nC-CP. Based on the results of pure hydrogel (Figure 6(b)), fibroblast was found to be viable within hydrogel as compared with control suggesting that the environment could sustain fibroblast growth and proliferation. In other words, nCur-CP could express good biocompatibility.

### 3.4. In Vivo Evaluation of Burn Wound Healing in Animal Model

#### 3.4.1. Examination Degree Burn Wound Model Design

In order to investigate the safety, efficacy action mechanism of the nCur-CP material in the mice second/third-degree burn injury model, we designed a custom-made device including steel rod (1 cm in diameter), water container, and electric induction. The container with water was heated by electric induction until the temperature of water reached 100°C. The steel rod was placed into this container at least 15 minutes before experimenting. The back hair of each rat was shaved with a shaving machine to obtain a smooth and hairless skin. This steel then was put on the back of the mice which were shaved with a shaving machine to obtain a smooth and hairless skin. In this study, various applied times of putting steel rod on the back of mice were examined through histological staining (H&E).

**Figure 1:**
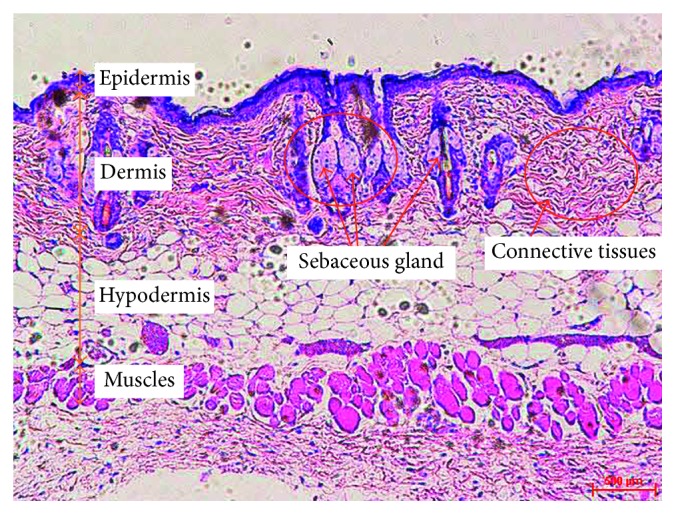
The histological images of normal skin tissue sections stained with H&E at 10x magnification (scale bar: 500⁡*μ*m).

**Figure 2:**
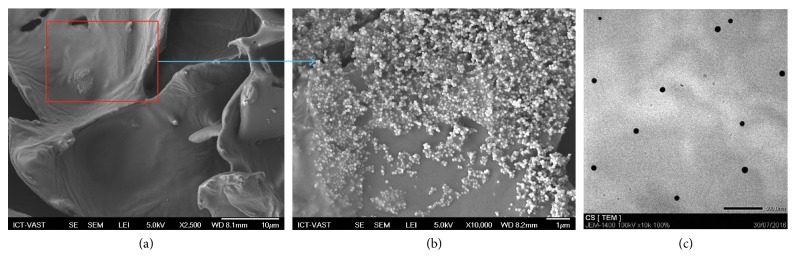
Representative scanning electron micrographs of the surface nCur-CP hydrogel composite (a, magnification: 2,500). Higher magnification images (b, magnification: 10,000) better show the presence of nanocurcumin on the surface. (c) Transmission electron microscopic (TEM) images of nanocurcumin (scale bar represents 500 nm).

**Figure 3:**
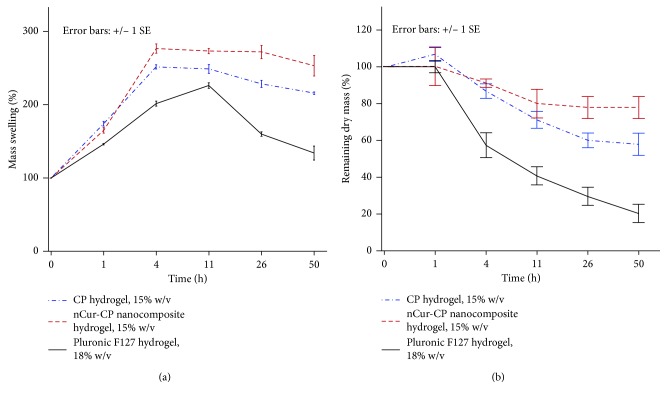
Swelling ratios and biodegradation pattern of the nCur-CP nanocomposite hydrogel (15% w/v), CP hydrogel (15% w/v), and Pluronic F127 hydrogel (18% w/v) as a function of time. The degradation rate was determined by the mass loss method (b), whereas the swelling behavior was investigated by the swollen mass method.

**Figure 4:**
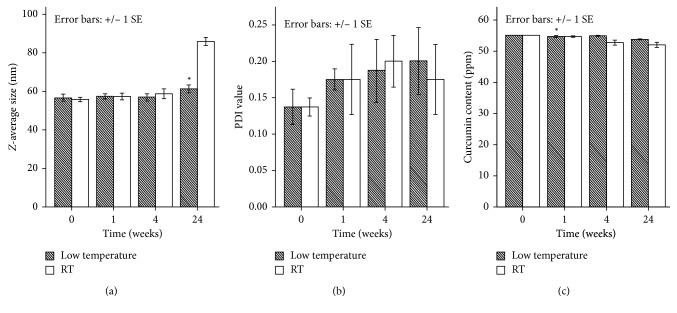
Reconstituted particle size (a), PDI (b), and content (c) of curcumin nanoparticles at 0, 1, 4, and 24 weeks of storage at different stability conditions: low temperature condition and ambient conditions (RT). Bars indicate SE (standard error of the estimate of the mean). From mean of 4 observations (*n*=4). Samples designated with the asterisk (^∗^) were significantly different (*p* < 0.05) as compared to initial time (one-way ANOVA was applied).

**Figure 5:**
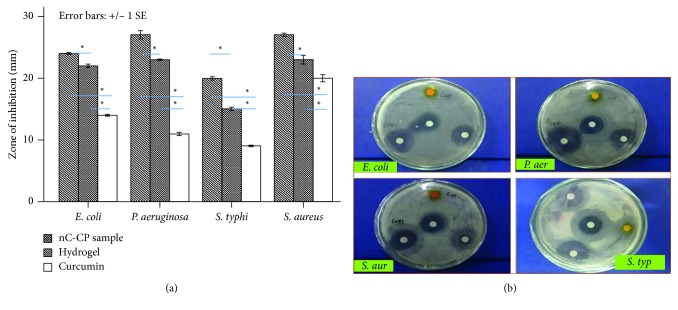
Antibacterial activity of hydrogel, nCur-CP, and raw curcumin solutions (a, b) against 4 bacterial strains. Bars indicate SE from mean of 3 observations (*n*=3). Samples designated with the asterisk (^∗^) were significantly different (*p* < 0.05) by ANOVA one-way comparison. Zone of inhibition of *Escherichia coli* (ATCC8739), *Salmonella typhimurium* (ATCC 14028), *Pseudomonas aeruginosa* (ATCC27853), and *Staphylococcus aureus* (ATCC 6538).

**Figure 6:**
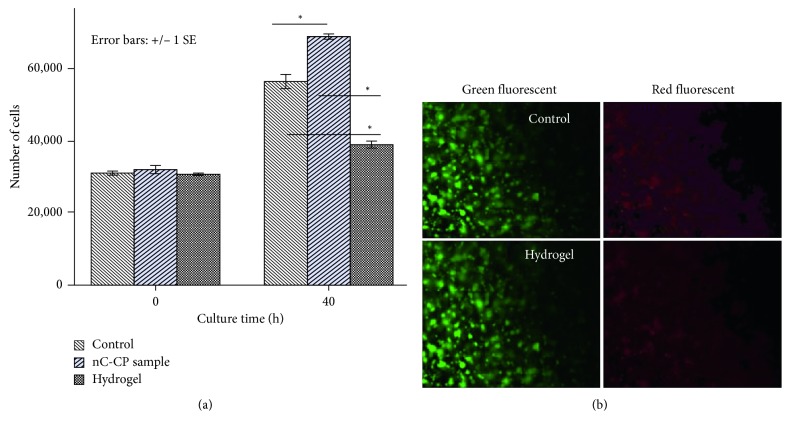
Effect of culture environment on HFF-1 cell proliferation (a) over 40 hours of culture. Results are presented as mean ± SE, and the asterisk (^∗^) indicates statistically significant differences at the given time points (*p* < 0.05). Representative micrographs taken of HFF-1 cultured on hydrogels and control over 1 week compatibility test. Cells are stained with live (green, calcein AM) and dead (red, ethidium homodimer-1) stains (b).

**Figure 7:**
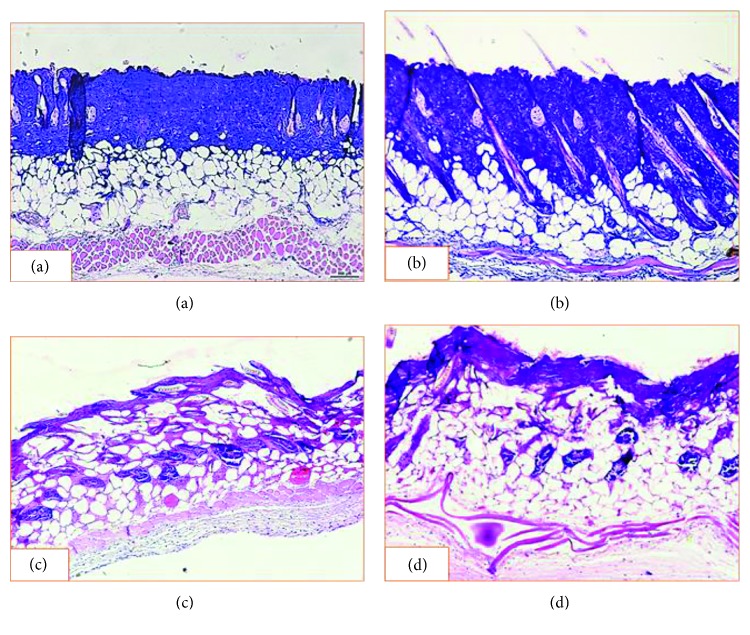
Photomicrograph of mice skin sections at various time applied steel rods which was heated at about 100°C: (a) 3 seconds; (b) 5 seconds; (c) 10 seconds; (d) 20 seconds. The skins stained with H&E, observed at 10x, bar = 500⁡*μ*m.

By comparing with the structure of normal skin section (Figure 1), the longer applied time led to the more damaged skin structure (Figure 7). At 3 seconds (Figure 7(a)), the significant damage was observed on epidermis and dermis layer. In contrast, application of a heat source of 100°C for 5 seconds caused injury to the superficial dermis but did not reliably cause full thickness (third-degree) burn injury (Figure 7(b)). At 10 seconds (Figure 7(c)), the epidermis layer and dermis layer were completely denatured as well as with damaged extending into the subcutaneous fat. In case of 20 seconds, not only the three layers of skin were damaged, but also the muscle layer was deformed. Therefore, use of 100°C for 3 seconds was chosen to generate second-degree burns whereas 10 seconds was selected to create third-degree burns.

#### 3.4.2. Evaluation of Second-Degree Burn Wound Healing in Mice Model

**Figure 8:**
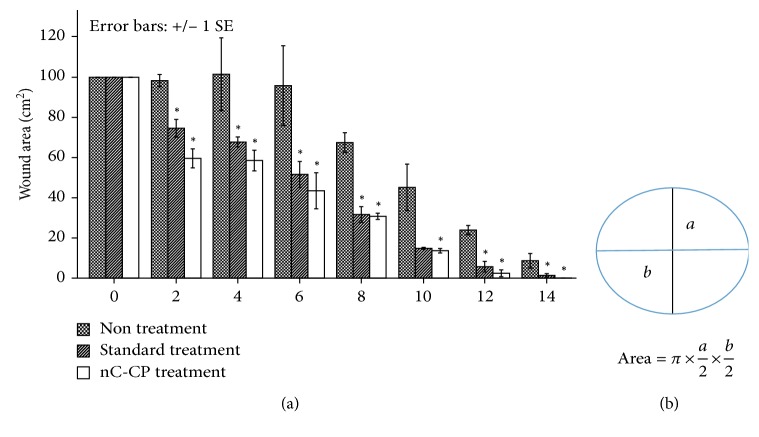
Results of second-degree burn wound size (a) in the wound-size reductive change between three groups. Measurement methods for wound size (b) based on Lyman [69]. As a protocol, measure the greatest length along the axial direction (b) and then the greatest width along the transverse direction (a) using a vernier caliper. Finally, multiply distances of length and width following the formula to obtain an estimate of the surface area in square millimeters (mm^2^). Values are statistically compared by ANOVA one-way and ANOVA two-way as well as Turkey's test. Results are presented as mean ± SE, and the asterisk (^∗^) indicates statistically significant differences at the given time points (*p* < 0.05).

Treatment efficacy of nCur-CP on mice with second-degree burn wound was investigated in comparison to other formulations such as hydrogel and standard treatment (Biafinecream used in clinical center for second degree burn treatment). Wound healing was followed and time to wound closure was measured as described in Figure 8(a). The mean wound surface area on day 0 of wounding was similar in all three groups (100 mm^2^). Wound closure occurred over time in all the three groups. As early as day 2, a difference was observed between the untreated and treated groups (*p* < 0.05). The difference in closure rates was much greater from day 4 to day 12 where the residual wound area was significantly larger in the nontreated group than in either of the other two groups (*p* < 0.05). The wounds in all of nCur-CP treatment were completely healed by day 14, while all wounds in the nontreatment were still unhealed at this time. In addition, although there was no significant different in statistic between standard treatment and nC-CP, expected the result at day 2, a little wound in standard treatment was healed at the end of the experiment period (14 days).

**Figure 9:**
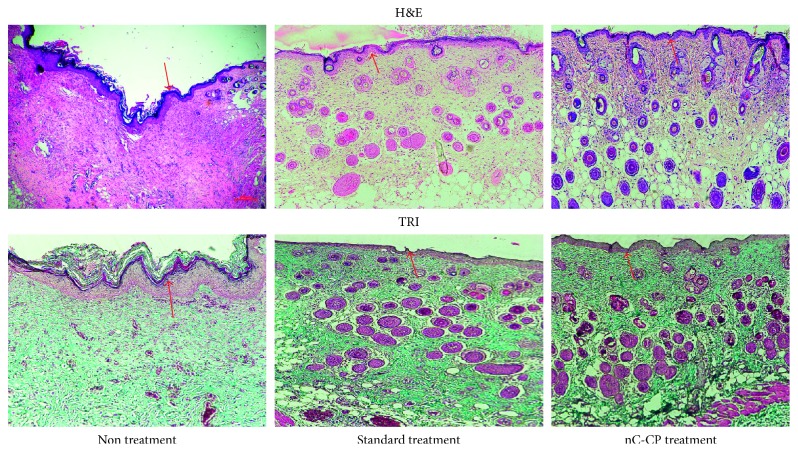
H&E and TRI staining images of second-degree burn wounds on day 14, observed at 10x, bar = 500⁡*μ*m. The red arrows indicate the regeneration of epidermis layer.

For additional confirmation of the quality and maturity of the healed tissues of nC-CP, standard treatment and nontreatment, histological staining was carried out. Full thickness sections of treated wounds, with highest healing potential, were collected and stained with hematoxylin and eosin (H&E) for microscopical examination of skin layers and stained with Masson's trichrome (TRI) for microscopical examination of connective tissue. All wounds in the three groups showed the presentation of epidermis layer (Figure 9). The skin section with nontreatment exhibited densely packed collagen fibers with parallel arrangement and high accumulation under well-formed thick epithelium. Moreover, numerous collagen bundles were observed in the reticular layer indicating uncompleted remodeling and still active in nontreatment group (*p* < 0.05). In contrast, wound in both treated groups showed almost normal skin structure with well-formed and differentiated epithelium and woven collagen fibers (*p* < 0.05). However, a more rapid remodeling was noted in the nanocomposite hydrogel-treated wounds. Specifically, the sebaceous gland had seemed the normal skin (Figure 1) while still prematurity was observed in standard treatment. Worthy mentioning that in all examined treated skin sections, no inflammatory cells were detected in three groups. These observations indicated that the nanocomposite was found to be superior in promoting the formation of the collagen and making re-epithelization in less time compared to standard treatment. This might further prove the combined effect of chitosan and curcumin in addition to the effect of this novel formulation to retain them inside the skin, speeding up the wound healing process with high probability for reducing scar formation.

#### 3.4.3. Evaluation of Third-Degree Burn Wound Healing in Mice Model

**Figure 10:**
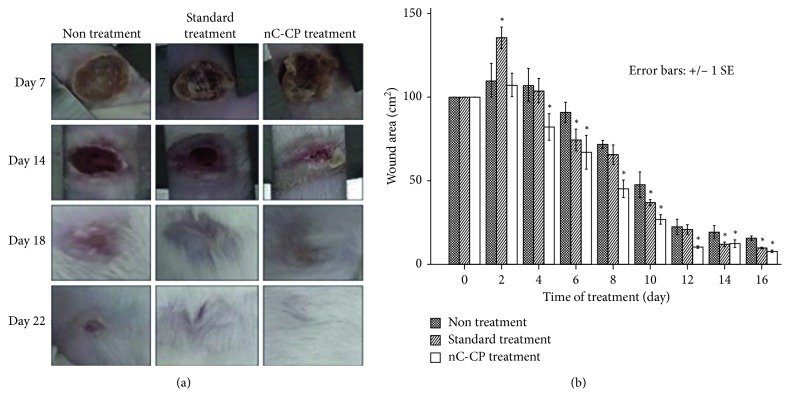
Digital photographical images of morphological wounds (a) and wound size reduction measurement for groups of nontreatment, standard treatment with silvirin (a commercial product for third-degree burn healing), and nCur-CP for third-degree burn wound (b). Values are statistically compared by ANOVA one-way and ANOVA two-way as well as Turkey's test. Results are presented as mean ± SE, and the asterisk (^∗^) indicates statistically significant differences at the given time points (*p* < 0.05).

**Figure 11:**
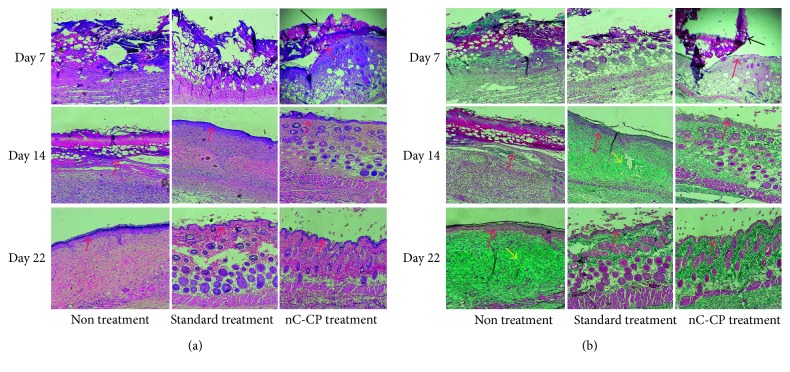
Histological examination of the change in third-degree burn wound over time (7, 14 and 22 days postwounding) with different treatment groups (a). The image of H&E staining and TRI staining (b). All the images were observed at 10x magnification and scale bar = 500. The black arrows and red arrows point out the crust and re-epithelialized layer, respectively. The yellow arrows indicate the formation of scar.

Third-degree burn wound symptoms in mice were observed both physically (Figures 10(a) and 10(b)) and histologically (Figure 11). The digital photographical images of morphological wounds indicated that the nC-CP-treated wound had the best healing rate after 21 days. For tracking wound contraction (Figure 10(b)), the wounding area had the same values at all groups at day 0. In addition, although the wounds received various treatments, all mean wound surface areas were reduced over time. Examining gross wound closure at day 2 showed the extension of wound surface areas in all three groups. The largest wounding area was in standard treated group compared with other groups (*p* < 0.05), while no significant different was detected between nCur-CP treated groups and nontreated group. This may be due to the effect of antibiotic agents used in the standard treatment. Between day 7 and day 12, the quickest closure rates were taken place in wounding of nCur-CP treatment (from 82.2 ± 17.35 mm^2^ to 12.2 ± 4.9 mm^2^, *p* < 0.05). Although only data through day 16 are presented in Figure 8(b), wounds in all of the standard treatment and nCur-CP treatment were completely healed by day 20, while untreated wounds were not completely healed by day 22 (Figure 10(a)).

As shown in Figure 11, during the proliferation stage at day 7, the epidermis was only presented on the nCur-CP treated group. It can be believed that these skin sections have completed the inflammation phase and have currently started the proliferation phase. In addition, the initial healing was detected in standard treatment group by the exhibition of granulocyte which was the signal of inflammation phase. New blood vessels were observed within the granulation tissue in the nCur-CP treated group and and blood had flowed into these cavities. At the same time, the crust was established on the surface of these skin sections. However, no crust was found on the wound surface of the other groups. Fourteen days after treatment, the scar formation showed in standard treatment, while the skin sections with nCur-CP treated group passed this stage. Identification of the neoepithelium on TRI-stained sections confirmed that standard treated wounds had an epithelial, while nC-CP-treated wounds were completely re-epithelialized at day 14. Besides, the growth rate and density of the regenerated blood vessels were significantly lower in these groups compared with the nCur-CP treated group (*p* < 0.05). Moreover, the elastic fibers were present in the periphery of nCur-CP treatment while only trace quantities of new collagen fibers were found standard treated wounds, suggesting that nCur-CP treated wounds were at a more advanced stage of remodeling compared to standard treated wounds at the same time. In this time, the untreated group also showed the epithelial gap. At the end of the treatment period, skin from treated standard and nC-CP-treated model was similar to the normal skin (Figure 1); that is, the epidermis appeared to be thinner than the epidermis of nontreatment mice. Additionally, there were some newborn hair follicles in the center of the wound; however, newborn hair follicles could not be observed in the center of the wound in the control or standard treated groups. Moreover, in the treated group, the sebaceous glands regenerated not only at the edge of the wound, but also in the center, whereas this phenomenon did not present in nontreated group. Based on this result, sampled on day 22, the nanocomposite samples significantly contributed to the acceleration of wound repair in impaired healing when compared to other groups in terms of both wound area surface and histological examination.

## 4. Conclusion

A new biofunctional injectable scaffold based on curcumin and thermal pluronic F127-grafted chitosan copolymer was studied and evaluated further for second- and third-degree burns treatment (Schematic 1). The injectable platform exhibited some structural characteristics that could be good for applications in tissue engineering. The nanocomposite hydrogel was more pronounced against wide range of bacteria (both positive gram and negative gram) when compared with the use of other single forms (CP hydrogel and curcumin). The obtained results indicated that the nanocomposite hydrogel is able to induce an appropriate response for the reconstruction of the skin after both - and third-degree burn injury models. It represents both an ideal material for tissue engineering and an ideal device such as easy to handle, transfer, and apply to wounds. The injectable nanocomposite materials should be studied further to produce a beneficial product for human healthcare.

## Figures and Tables

**Scheme 1:**
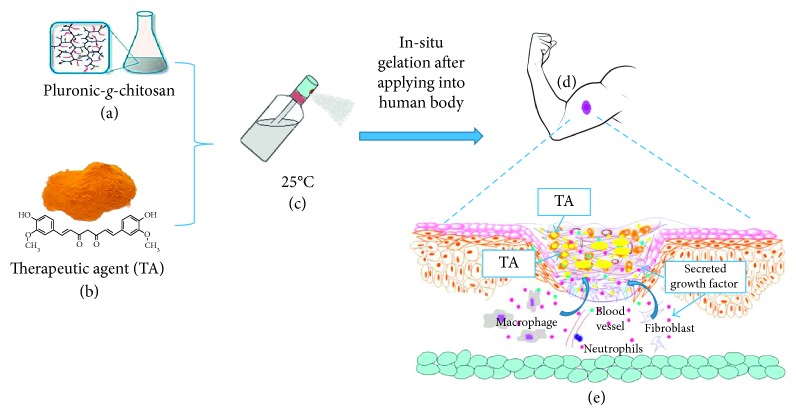
Proposal process of nCur-CP nanocomposite hydrogel for burn wound healing. (a) CP is synthesized through the conjugation of pluronic F127 and amide groups on chitosan at the specific ratios (pluronic F127 : chitosan = 15 : 1 in weight). (b) Curcumin in process into nanoform by the help of CP resulting in the formation of nCur-CP nanocomposite hydrogel (c) which has the sol-gel transition near the temperature of human body; thus, this solution is in gelation after contacting with wound surface (d). (e) nCur-CP nanocomposite hydrogel could be a promising therapeutic strategy for the management of burn wound.

**Table 1:** MIC of raw curcumin, hydrogel, and nCur-CP against different microbes. In each concentration of nC-CP, the content of curcumin was determined based on the content of curcumin in the stock of nCur-CP and series dilution.

Organisms	MIC		
	Raw curcumin (ppm)	Hydrogel (mg/ml)	nCur-CP(mg/ml)
*E. coli*	1.6	18.75	3.0 (0.32 ppm curcumin)
*S. typhimurium*	4	37.5	4.5 (0.48 ppm curcumin)
*P. aeruginosa*	2	9.0	0.75 (0.08 ppm curcumin)
*S. aureus*	0.96	15	1.5 (0.16 ppm curcumin)
